# Coffee Intake Decreases Risk of Postmenopausal Breast Cancer: A Dose-Response Meta-Analysis on Prospective Cohort Studies

**DOI:** 10.3390/nu10020112

**Published:** 2018-01-23

**Authors:** Alessandra Lafranconi, Agnieszka Micek, Paolo De Paoli, Sabrina Bimonte, Paola Rossi, Vincenzo Quagliariello, Massimiliano Berretta

**Affiliations:** 1 The Research Centre on Public Health, University Milano-Bicocca, 20900 Monza, Italy; alessandra.lafranconi@unimib.it; 2 Department of International Health, FHML, CAPHRI, Maastricht University, 6229 Maastricht, The Netherlands; 3 Department of Epidemiology and Population Studies, Jagiellonian University Medical College, 31008 Krakow, Poland; agnieszka.micek@uj.edu.pl; 4 Scientific Directorate, National Cancer Institute-IRCCS, 33081 Aviano, Italy; pdepaoli@cro.it; 5 Division of Anesthesia and Pain Medicine, Istituto Nazionale Tumori-IRCCS-“Fondazione G. Pascale”, 80131 Naples, Italy; s.bimonte@istitutotumori.na.it; 6 Department of Biology and Biotechnology (DBB) “L. Spallanzani”, University of Pavia, 27100 Pavia, Italy; paola.rossi@unipv.it; 7 Department of Abdominal Oncology, National Cancer Institute, IRCCS-Foundation G. Pascale, 80131 Naples, Italy; quagliariello.enzo@gmail.com; 8 Department of Medical Oncology, National Cancer Institute-IRCCS, 33081 Aviano, Italy

**Keywords:** coffee, caffeine, breast cancer, receptor, postmenopausal, dose-response, meta-analysis

## Abstract

**Aim:** A dose-response meta-analysis was conducted in order to summarize the evidence from prospective cohort studies regarding the association between coffee intake and breast cancer risk. **Methods:** A systematic search was performed in electronic databases up to March 2017 to identify relevant studies; risk estimates were retrieved from the studies and linear and non-linear dose-response analysis modelled by restricted cubic splines was conducted. A stratified and subgroup analysis by menopausal and estrogen/progesterone receptor (ER/PR) status, smoking status and body mass index (BMI) were performed in order to detect potential confounders. **Results:** A total of 21 prospective studies were selected either for dose-response, the highest versus lowest category of consumption or subgroup analysis. The dose-response analysis of 13 prospective studies showed no significant association between coffee consumption and breast cancer risk in the non-linear model. However, an inverse relationship has been found when the analysis was restricted to post-menopausal women. Consumption of four cups of coffee per day was associated with a 10% reduction in postmenopausal cancer risk (relative risk, RR 0.90; 95% confidence interval, CI 0.82 to 0.99). Subgroup analyses showed consistent results for all potential confounding factors examined. **Conclusions:** Findings from this meta-analysis may support the hypothesis that coffee consumption is associated with decreased risk of postmenopausal breast cancer.

## 1. Introduction

Breast cancer is the most frequently diagnosed cancer and among the leading causes of cancer death among females [1,2,3]. Worldwide and European estimates of women with a diagnosis of breast cancer occurring in the last 5 years were over 6.2 and 1.8 million, respectively, in 2012 [4]. Significant improvements in early diagnosis and treatment have led to decreased mortality in the last two decades [5,6]. However, evaluating potential risk factors and improving preventive actions is needed in order to decrease the global burden of such disease.

A recent summary of scientific literature provided insightful the evidence of the potential benefits of coffee on human health [7]. Most of evidence relied on observational prospective cohort studies, suggesting that moderate-to-high coffee can be overall associated with lower risk of all-cause, cardiovascular and cancer mortality compared to lower consumption [8]. These protective effects are likely to be mediated by coffee active compounds, including but not limited to, coffee polyphenols, which have been shown to have anti-diabetic, anti-carcinogenic, anti-inflammatory and anti-obesity properties [9,10,11]. Among others, coffee consumption has been hypothesized to affect the risk of female cancers (breast, endometrial and ovarian cancers) and particularity the risk of breast cancer in post-menopausal women. A recent analysis of dietary patterns of women participating in the Nurses’ Health Study II pointed out that a low intake of green leafy vegetables, cruciferous vegetables and coffee during adolescence and early adulthood may increase the incidence of premenopausal breast cancer, thus posing the basis for further research on long-term associations and cumulative effects [12].

Previous quantitative evidence syntheses on breast cancer risk often reported contrasting results, either when assessing the risk estimates for extreme categories of consumption and in a dose-response manner [13,14,15,16,17]. For instance, the meta-analysis carried out by Li and colleagues—on 16 cohort and 10 case-control studies—found a borderline significant association (RR: 0.96; 95% CI: 0.93–1.00) when comparing highest versus lowest coffee consumption; results limited to cohort studies were not significant. Interestingly, a significant inverse association between coffee drinking and breast cancer risk was documented in women without oestrogen receptor (ER-negative subgroup) [18]. Similarly, Jiang and colleagues analysed 20 case-control and 17 cohort studies and reported a borderline association with breast cancer risk of the highest coffee consumption category compared to the lowest (RR: 0.97; 95% CI: 0.93–1.00); yet, a significant inverse association was present in postmenopausal women and in women with breast related cancer antigen 1 (BRCA1) mutations (the latter being highlighted as strong association) [19]. Therefore, coffee consumption might have a protective role for specific subgroups of individuals (i.e., based on receptor and menopausal status) [18,19]; Since the latest meta-analyses from Jing et al. and Li et al., several new prospective studies have been published on the association between coffee intake and breast cancer. Thus, the aim of this study was to update current evidence on the association between coffee consumption and risk of breast cancers, analysing results obtained from prospective studies only, in order to summarize the evidence and provide new insights on potential effect modification of putative confounding factors.

## 2. Methods

We followed Meta-Analysis of Observational Studies in Epidemiology (MOOSE) protocols throughout preparing background, search strategy, methods and reporting the results, discussion and conclusion of meta-analysis (Table S1).

### 2.1. Search Strategy

Articles were retrieved by searching two different electronic databases (PubMed (http://www.ncbi.nlm.nih.gov/pubmed/) and EMBASE (http://www.embase.com/) and were limited to publications in English language between the earliest available online indexing year and March 2017. The following search strategy based on the conjunction of the three terms: (i) coffee OR caffeine OR beverages AND (ii) breast AND (iii) cancer OR carcinoma OR neoplasm (Table S2) was adopted. Titles and abstracts of all identified studies were independently reviewed by two authors. Based on recent guidelines proposed to draft the highest level of evidence in nutritional science [20], eligibility criteria for study inclusion in the meta-analysis were based on the following criteria: (1) a prospective design; (2) coffee consumption as the exposure of interest; (3) incidence of breast cancer as the outcome; (4) the measure of association (relative risk or hazard ratio) with 95% confidence interval provided for 3 or more quantitative categories of coffee consumption. Hand searching the reference lists of obtained manuscripts was also performed to find additional studies not previously detected. In the case of duplicated published cohorts, the one with the largest number of cases/entire cohort or with the longest follow-up for endpoint of interest was included.

### 2.2. Data Extraction

Using a standardized extraction form, data were abstracted from all identified studies. The following information was obtained from each article: (1) first author name; (2) year of publication; (3) study cohort name; (4) country; (5) sex of participants; (6) age range of the study population at baseline; (7) categories of coffee consumption; (8) type of coffee; (9) follow-up period; (10) distribution of cases and person-years/number of participants, across categories of exposure; (11) relative risks or hazard ratios, with 95% CIs for all categories of exposure; (12) covariates used in adjustments. Extraction of data was conducted independently by two authors. Discrepancies were resolved through a consensus discussion, The Newcastle-Ottawa Quality Assessment Scale was used to assess the quality of included studies [21].

### 2.3. Statistical Analysis

For the purpose of this meta-analysis, relative risks (RRs) or hazard ratios (HRs) with 95% confidence intervals (CIs) for all categories of coffee consumption were extracted based on the most fully adjusted models. Pooled effects were assessed by random-effect meta-analyses in which RRs and HRs were treated as equivalent measure of risk [22] and the term of the relative risk refer to both of them. Heterogeneity was assessed using *I*^2^ statistic and the Cochran’s Q test. The *I*^2^ statistic represented the amount of total variation that could be attributed to heterogeneity and its values ≤25%, 25–50%, 50–75% and >75% indicated no, small, moderate and significant heterogeneity, respectively. The *P* values of Q test of less than 0.1 were accepted as statistically significant. The relationship between coffee consumption and risk of breast cancer was firstly determined by highest versus lowest analysis. The stability of the results was assessed through a sensitivity analysis in which one study at a time was excluded. Potential confounders or effect modifiers and sources of heterogeneity were verified in subgroup analysis and publication bias was tested visually by detecting asymmetry of funnel plots.

Based on retrieved data (amount of exposure, distributions of cases and person-years or number of participants and RRs/HRs with 95% CIs) for each category (at least three) of coffee consumption, a dose-response meta-analysis was performed. Within the studies, the mean or median intake, alternatively the midpoint of the range of intake of coffee consumption was assigned to the corresponding RR/HR with the 95% CI. Right-unbounded highest categories of exposure were assumed to have the same width as the adjacent one. Both linear and non-linear dose-response relationship between coffee intake and risk of total and postmenopausal breast cancer was assessed by random-effect meta-analysis performed in two stages. In the first stage, the generalized least-squares (GLS) method reported by Greenland and Orsini was implemented and study-specific coefficients were calculated based on retrieved data across categories of coffee consumption and taking into account the intraclass (within study) correlation of RRs/HRs [23,24]. Non-linear dose-response analysis was modelled by restricted cubic splines with 3 knots at fixed percentiles (25%, 50% and 75%) of the distribution [25]. In the second step of the random-effect meta-analysis summary statistics from each study were combined. The between-study variance in linear dose-response meta-analysis and the between-study covariance matrices in non-linear dose-response meta-analysis was assessed by DerSimonian and Laird estimator or multivariate extension of the method of moments, respectively. *P*-value for non-linearity was calculated by testing the value of the coefficient of the second spline of zero. All analyses were performed with R software version 3.0.3, to conduct dose-response meta-analysis the package dosresmeta was used (Development Core Team, Lucent Technologies, Vienna, Austria).

## 3. Results

### 3.1. Study Characteristics

**Figure 1:**
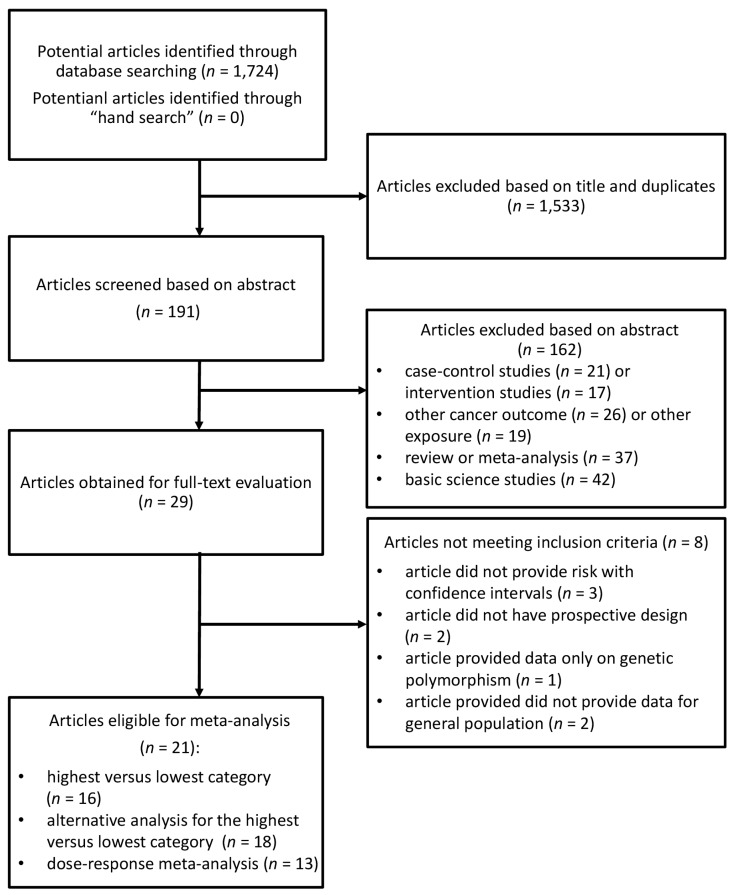
Flow chart of selection of studies reporting on the association between coffee consumption and breast cancer risk.

The systematic search identified 1724 studies, of which 1533 were excluded after reviewing the title and 162 after reviewing the abstract (Figure 1). Of the 29 publications selected for evaluation of full-text article, 8 were excluded for the following reasons: (1) article did not provide risk with confidence intervals; (2) article did not have prospective design; (3) article provided data only on genetic polymorphism; (4) article did not provide data for general population.

For the meta-analysis on the association between coffee consumption and breast cancer risk 21 studies were eligible [26,27,28,29,30,31,32,33,34,35,36,37,38,39,40,41,42,43,44,45,46]. Several cohorts, namely VIP (Västerbotten Intervention Programme), NOWAC (Norwegian Women and Cancer), E3N (Etude Epidémiologique auprès de femmes de la Mutuelle Générale de l’Education Nationale) and EPIC-NL (European Prospective Investigation into Cancer and Nutrition—The Netherlands) [26,29,40,42] were excluded from the main analysis, as part of theirs cases are included in the multicentre study EPIC [27]. However, an alternative analysis was performed by including these cohorts and excluding EPIC study. Two articles were used only for subgroup analysis [33,41]. Studies eligible for the main analysis comprised 1,068,098 participants and 36,597 breast cancer cases. Selected characteristics of the studies included in the meta-analysis are described in Table 1. Eight studies provided relative risk for postmenopausal [27,28,30,31,33,37,43,45] and for premenopausal status [27,28,32,37,41,43,45,46]. Seven studies were conducted in North America [28,30,31,32,34,37,46], 6 in Europe [27,35,36,39,43,45] and 2 in Asia [38,44]. The follow-up in prospective cohort studies ranged from about 5 to 26 years.

### 3.2. Summary Relative Risk for the High vs. Lowest Category of Coffee Consumption

**Figure 2:**
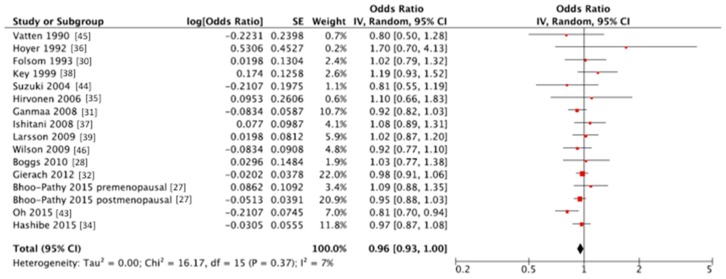
Forest plot of summary relative risks (RRs) of breast cancer for the highest versus lowest (reference) category of coffee consumption. SE = standard error; CI = confidence interval; IV = instrumental variable.

The summary RR of breast cancer for highest vs. lowest category of coffee consumption was RR = 0.96, 95% CI: 0.93, 1.00 with small heterogeneity *I*^2^ = 7%, *p* = 0.37 (Figure 2, Table 2); no publication bias was found after visual inspection of funnel plot (Figure S1). Two studies [36,38] were at higher risk of bias and provided very small heterogeneity to the overall analysis, despite no specific reasons have been identified; we may hypothesize that quality of the data was not optimal since the studies were not conducted specifically on coffee as variable of interest but this is only speculative and no data can support this explanation. Since several cohorts (VIP, NOWAC, E3N, EPIC-NL) share the cases with EPIC multicentre study an alternative analysis by excluding EPIC study and including the others was performed. The summary RR of breast cancer for highest vs. lowest category of coffee consumption in alternative analysis was RR = 0.96, 95% CI: 0.93, 1.00 with no evidence of heterogeneity *I*^2^ = 0%, *P* = 0.48; indicating the stability of the results. In the stratified analysis, we found a significant inverse association between coffee consumption and breast cancer risk among postmenopausal women (RR = 0.92, 95% CI: 0.88, 0.98 with no evidence of heterogeneity *I*^2^ = 0%, *p* = 0.57). Stratified analyses were carried out for receptor status and BMI: none of them showed a significant association between coffee intake and cancer risk; yet, similar point estimates and confidence intervals were found for the receptor-negative group and for the overweight and obese group. Interestingly, results were not influenced by coffee type (caffeinated versus decaffeinated), nor for time of follow-up. Adjusted analyses were carried out for smoking, alcohol intake, physical activity and education, without evidence of effect modification.

### 3.3. Dose-Response Meta-Analysis

**Figure 3:**
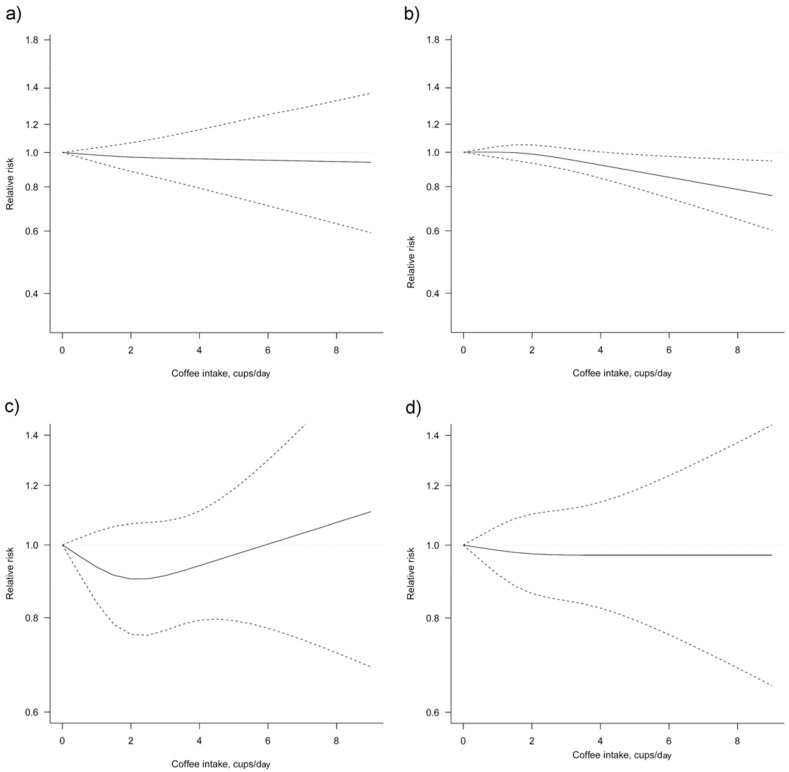
Dose-response association between coffee consumption and breast cancer risk (**a**) non-linear, total analysis; (**b**) non-linear, postmenopausal; (**c**) non-linear, receptor status ER-/PR-; (**d**) non-linear, BMI ≥ 25 kg/m^2^. Solid lines represent relative risk, dashed lines represent 95% confidence intervals.

Thirteen studies [26,28,29,31,32,34,37,38,39,40,42,43,45] were eligible for dose-response meta-analysis of prospective cohort studies on coffee consumption and breast cancer risk. Six studies [32,34,41,42,43,45] provided risk estimates for postmenopausal woman only. In linear dose-response meta-analysis a significant association between coffee consumption and breast cancer risk was found (Figure 3, Table 3). Compared with no coffee consumption, the pooled relative risks for breast cancer were 0.99, 95% CI: 0.98, 1.00 for one cup/day, 0.98, 95% CI: 0.96, 0.99 for two cups/day, 0.97, 95% CI: 0.94, 0.99 for three cups/day, 0.96, 95% CI: 0.93, 0.99 for four cups/day, 0.95, 95% CI: 0.91, 0.98 for five cups/day, 0.93, 95% CI: 0.89, 0.98 for six cups/day and 0.92, 95% CI: 0.88, 0.98 for seven cups/day. When taking into account postmenopausal woman only, the association between coffee consumption and risk of breast cancer was stronger.

## 4. Discussion

The present dose-response meta-analysis, which included 13 prospective studies and followed over 1 million people, did not show a significant association between coffee consumption and overall breast cancer risk in the non-linear model. However, an inverse relationship has been found when the analysis was restricted to post-menopausal women (RR 0.92, 95% CI: 0.88–0.98).

The meta-analysis performed by Li and colleagues [18], on 16 cohort and 10 case-control studies, showed a borderline significant inverse association between coffee intake and the risk of breast cancer (RR: 0.96, CI 95%: 0.93–1.00 for highest versus lowest analysis; RR: 0.98, CI 95%: 0.97–1.00 for an increment of 2 cups per day). Statistical significance was reached only for those women without oestrogen receptor (ER-negative, RR: 0.81, 95% CI: 0.67–0.97). In our study, such finding was not confirmed. The work carried out by Jiang and colleagues [19], which included 17 prospective and 20 case-control studies, found no significant association between coffee consumption and breast cancer risk (highest versus lowest analysis: RR: 0.98, CI 95% 0.95–1.02; dose-response analysis: RR: 0.98, 95% CI: 0.92–1.05 for an increment of 2 cups per day). Such relationship became significant for post-menopausal women, with a stronger inverse relationship for those with BRCA1 mutation. Overall results are mostly confirmed in our meta-analysis, even though we included a higher number of individuals and performed alternative analyses to avoid overlap of cohorts. However, while our analysis confirmed a statistically significant relationship between coffee intake and breast cancer in post-menopausal women, we were not able to draw insights on the role of BRCA status (evidence derived from case-control studies, not eligible for our meta-analysis). Moreover, similarly to our study, the meta-analysis of Jiang and colleagues differentiated the analysis according to follow-up duration and found similar results between studies with long (>10) and short (<10 years) follow-up.

Several studies associated coffee consumption with health benefits, including decreased risk of cancers (e.g., colorectal, endometrial and prostate cancers) [7,13,14]. Several compounds have been considered responsible for such potential protective effects, including polyphenols (such as chlorogenic acids), diterpens (such as cafestol and kahweol) but also melanoidins (generated during the roasting process) and trigonelline [47,48,49,50]. Data from in vitro and in vivo studies suggest that coffee could interfere with different stages of the cancerous process, including induction of DNA damage caused by pro-carcinogens and reactive oxygen species (ROS), activation of proto-oncogenes and inactivation of onco-suppressor genes, loss of apoptosis and growth control, induction of angiogenesis and consequent metastatic process [11].

Our study revealed significant results for post-menopausal women. Several mechanisms have been proposed to act specifically in the etiopathogenesis of female cancers: for instance, caffeine and coffee intake have been inversely associated with free estradiol levels in premenopausal women, either directly or indirectly (relationship mediated by the sex hormone-binding globulin-SHBG) [51,52,53]. SHBG, which is the major carriers of sex-steroids, lowers the circulating free levels of oestrogens and the positive association between coffee intake and SHBG has been documented in post-menopausal women as well [54]. Similarly, coffee has been associated with the inhibition of CYP19, or aromatase, the enzyme converting androgens into oestrogens [51]. Circulating oestrogens are well-established risk factors for breast cancer [55].

Despite the complexity of the described mechanisms, it is crucial to understand the pathways through which coffee may decrease risk of cancer. Our finding, showing a similar association for caffeinated and decaffeinated coffee, may suggest a limited role of caffeine on breast cancer risk (the literature highlights the role of caffeine on neurotransmitters, therefore on neurological, cardiorespiratory and gastrointestinal diseases) [7]. It is therefore more plausible that the action on carcinogenesis is mediated by other coffee compounds [11]. The beneficial effects may be related to the antioxidant ability, as coffee is one of the major contributor to dietary antioxidant intake worldwide [56,57]. However, based on the observational nature of the studies involved in this research, we cannot rule out the possibility of existence of confounding factors or effect modifiers that can indirectly explain the potential benefits of coffee toward breast cancer risk. For instance, one of the stronger evidence regarding coffee consumption regards liver and, more generally, metabolic health [58]. Several studies showed a better metabolic status occurring in individuals characterized by high consumption of coffee [10,59,60,61,62,63,64,65,66,67,68]. We found no difference in the relationship between coffee intake and breast cancer risk according to BMI; nevertheless, obesity is a well-recognized risk factor for breast cancer and hormonal impairment consequent to excess body weight may be inversely associated with consumption of coffee [69,70]. The metabolic syndrome, also known as insulin resistance syndrome, is a multifactorial disease well related to breast cancer risk of incidence and recurrence [71,72] with a crucial role in the activation of many endocrine and immune factors linked to breast cancer cell proliferation, survival and chemo-resistance. Among different nutritional approach aimed to revert metabolic syndrome in breast cancer patients, it was recently shown that consumption of coffee in adults up to three cups a day reduces the risk of type-2 diabetes, the metabolic syndrome as well as non-alcoholic fatty liver disease (NAFLD) [58,73]. Several studies showed that the association is rather stronger with the overall metabolic status than with individual components of the syndrome [67,74]. A crucial endocrine role in breast cancer risk and progress may be related to low levels of adiponectin (having anti-cancer effect) and high levels of leptin (having insulin and IGF1-like effects in cancer cells) in the blood [75,76]; interestingly, in a cross-sectional study, it was demonstrated that coffee consumption has a significant positive associations with adiponectin and inverse associations with leptin as well as with the inflammatory marker high sensitivity C-reactive protein (hs-CRP) indicating interesting endocrine and metabolic effects of coffee consumption [77].

In our study, we investigated the possible role of confounding factors in determining the association between coffee consumption and breast cancer risk. It has been reported that smoking status, education, physical activity and BMI, may cluster together with dietary choices that may affect cancer risk (i.e., consumption of meat, fruit and vegetable) [78]. Besides BMI, for which some studies provided specific RRs and we were able to perform the aforementioned stratified risk analysis, in our study the results of studies grouped according to adjustment for specific variables, such as smoking status, education and physical activity, did not weakened the results, rather they shortened CIs despite yet not significant, suggesting that better designed cohort studies properly adjusting for potential confounding factors may enhance the overall quality of comprehensive quantitative synthesis and leading to significant results toward lower risk of breast cancer associated with higher consumption of coffee.

Another indirect confounding effect may be related to alcohol consumption: alcohol is another known risk factor for breast cancer through the alteration of biological pathways associated to hormone levels, the production of carcinogens through metabolism of ethanol and the inhibition of the one-carbon metabolism and nucleotide biosynthesis [79,80]. Some descriptive studies on beverage drinking patterns suggest that increased coffee/tea consumption might be associated with alcohol intake [81,82,83,84,85,86]. However, our results showed that studies adjusting for alcohol intake provided lower risk estimates for the association between coffee and breast cancer risk, suggesting that adjustment for such lifestyle factors is crucial when exploring the potential effects of coffee on health. Coffee drinking has been associated with smoking habits, which in turn may exert detrimental effects on health [87]; in contrast, coffee might be a part of an overall healthier dietary pattern, which in turn might be associated with lower risk of cancer due to a synergistic effect of several components rather than an individual food or beverage [88]. More in-depth studies with further evaluation of genetic parameters are ongoing but additional research is needed to conclude a causal relation between coffee consumption and risk of cancer.

Besides the aforementioned limitations related to the original design of the studies included in this meta-analysis, the presented results should be considered in light of some other limitations. No data on methods of preparation have been provided in the studies, leading to possible heterogeneity of results due to differences in quantity and quality of bioactive compounds [89]. Finally, due to the observational nature of the studies included in the meta-analysis we cannot exclude the possibility of recall bias and reverse causation.

## 5. Conclusions

In conclusion, overall we observed no significant association between coffee intake and breast cancer risk but coffee consumption may represent a protective factor for post-menopausal breast cancer risk. Further evidences taking into account population subsets and specific strata are extremely needed to corroborate the retrieved associations.

## Figures and Tables

**Table 1:** Selected characteristics of the studies included in the meta-analysis. RR = relative risk. HR = hazard ratio.

Author, Year	Cohort Name, Country	Years of Study, Follow-Up	Cases; Total Population	RR (95%CI) for Highest vs. Lowest Category of Coffee Consumption	Adjustments
Vatten, 1990 [45]	National Health Screening Service, Norway	1974-NR, 12 years (mean)	155; 14,593	≥7 cups/day vs. ≤2 cups/day; RR = 0.80 (0.50, 1.40)	Age.
Hoyer, 1992 [36]	The Glostrup Population Studies, Denmark	1964–1990, 26 years (maximum)	51; 5207	≥7 cups/day vs. ≤2 cups/day; RR = 1.70 (0.70, 4.30)	Social class, age at menarche, menopause status, number of full-term pregnancies, height, weight, BMI, alcohol, smoking.
Folsom, 1993 [30]	Iowa Women’s Health Study, USA	1986–1990, 5 years	580; 34,388	≥4 cups/day vs. 0 cups/day; RR = 1.02 (0.79, 1.30)	Age, waist/hip ratio, number of live births, age at first live birth, age at menarche, FHBC (family history of breast cancer), family history × waist/hip ratio and family history × number of live births.
Key, 1999 [38]	The Radiation Effects Research Foundation’s Life Span Study, Japan	1969–1993, 24 years (maximum)	427; 34,759	≥5 cups/week vs. ≤1 cups/week; RR = 1.19 (0.93, 1.52)	Age, calendar period, city, age at time of bombings, radiation dose.
Michels, 2002 [41]	Swedish Mammography Cohort, Sweden	1987–1997, 9.5 years	1271; 59,036	≥4 cups/day vs. ≤1 cup/day; HR = 0.94 (0.75, 1.28)	Age, family history of breast cancer, height, BMI, education, parity, age at first birth, alcohol consumption, total caloric intake.
Suzuki, 2004 [44]	Cohort I-Cohort II, Japan	1984-NR Cohort I, 9 years;1990-NR Cohort II, 7 years	103; 8799 Cohort I; 119; 11,288 Cohort II	≥1 cup/day vs. never; RR = 0.81 (0.55, 1.18)	Age, types of health insurance, age at menarche, menopausal status, age at first birth, parity, mother’s history of breast cancer, smoking, alcohol drinking, BMI, consumption frequencies of black tea.
Hirvonen, 2006 [35]	Supplementation en Vitamines et Mineraux Antioxydants (SU.VI.MAX), France	1994–2002, 6.6 years (median)	95; 4396	≥3 cups/day vs. ≤1 cup/day; RR = 1.10 (0.66, 1.84)	Age, smoking, number of children, use of oral contraception, family history of breast cancer, menopausal status.
Ganmaa, 2008 [31]	National Health Service I, USA	1976–2002, 22 years	5272; 85,987	≥4 cups/day vs. <1 cup/month; RR = 0.92 (0.82, 1.03)	Age months, smoking status, BMI, physical activity, height, alcohol intake, family history of breast cancer in mother or a sister, history of benign breast disease, menopausal status, age at menopause, use of hormone therapy, age at menarche, parity and age at first birth, weight change after 18, duration of postmenopausal hormone use, tea intake.
Ishitani, 2008 [37]	Women’s Health Study, USA	1992–2004, 10 years (average)	1188; 38,432	≥4 cups/day vs. almost never; RR = 1.08 (0.89, 1.30)	Age, randomized treatment assignment, BMI, physical activity, total energy intake, alcohol intake, multivitamin use, age at menopause, age at menarche, age at first pregnancy lasting ≥6months, number of pregnancies lasting ≥6months, menopausal status, postmenopausal hormone use, prior hysterectomy, prior bilateral oophorectomy, smoking status, family history of breast cancer in mother or a sister, history of benign breast disease.
Larsson, 2009 [39]	Swedish Mammography Cohort (SMC), Sweden	1987–2007, 17.4 years (mean)	2952; 61,433	≥4 cups/day vs. <1 cup/day; RR = 1.02 (0.87, 1.20)	Age, education, BMI, height, parity, age at first birth, age at menarche, age at menopause, use of oral contraceptives, use of postmenopausal hormones, family history of breast cancer and intakes of total energy, alcohol and tea.
Wilson, 2009 [46]	National Health Service II, USA	1991–2005, 14 years	1179; 90,628	≥3 cups/day vs. <1 cup/day; RR = 0.92 (0.77, 1.11)	BMI, height, OC use, parity and age at first birth, age at menarche, family history of breast cancer, history of benign breast disease, smoking, physical activity, animal fat, glycaemic load, alcohol and energy.
Bhoo-Pathy, 2010 [26]	European Prospective Investigation into Cancer and Nutrition (EPIC-NL), Netherlands	1993–2007, 9.6 years (average)	681; 27,323	>5 cups/day vs. <1 cup/day; HR = 0.94 (0.72, 1.24)	Age, smoking status, educational status, BMI, alcohol intake, energy intake, energy-adjusted saturated fat intake, energy-adjusted fibre intake, tea intake, physical activity level, ever prior use of oral contraceptives, presence of hypercholesterolemia, family history of breast cancer, age at menarche, parity and cohort.
Boggs, 2010 [28]	Black Women’s Health Study, USA	1995–2007, 12 years	1268; 52,062	≥4 cups/day vs. never; RR = 1.03 (0.77, 1.39)	Age, energy intake, age at menarche, BMI at age 18, family history of breast cancer, education, geographic region, parity, age at first birth, oral contraceptive use, menopausal status, age at menopause, female hormone use, vigorous activity, smoking status, alcohol intake.
Nilsson, 2010 [42]	Västerbotten Intervention Programme (VIP), Sweden	1992–2007, 15 years (maximum)	587; 32,178	≥4 cups/day vs. <1 cup/day; HR = 0.92 (0.68, 1.25)	Age, sex, BMI, smoking, education, recreational physical activity.
Fagherazzi, 2011 [29]	Etude Epidémiologique auprès de femmes de l’Education Nationale (E3N), France	1993–2005, 11 years (median)	2868; 67,703	>3 cups/day vs. never; HR = 1.02 (0.90, 1.16)	Age, total energy intake, ever use of oral contraceptives, age at menarche, age at menopause, number of children, age at first pregnancy, history of breast cancer in the family and years of schooling, current use of postmenopausal hormone therapy (for postmenopausal women only), personal history of benign breast disease, menopausal status, BMI.
Gierach, 2012 [32]	NIH-AARP Diet and Health Cohort Study, USA	1995–2006, 9.8 years (average)	9915; 198,404	≥4 cups/day vs. never; RR = 0.98 (0.91, 1.07)	Age, race/ethnicity, education, BMI, smoking status and dose, alcohol, proportion of total energy from fat, age at first live birth, menopausal hormone therapy use, history of breast biopsy, family history of breast cancer.
Bhoo-Pathy, 2015 [27]	European Prospective Investigation into Cancer and Nutrition (EPIC), Multicentre	1992–2010, 11 years (average)	10,198; 335,060	Highest quartile vs lowest quartile; HR = 1.00 (0.98, 1.03) for premenopausal women; HR = 0.99 (0.98, 0.99) for postmenopausal women	Age at menarche, ever use of oral contraceptives, age at first delivery, ever breastfeeding, smoking status, educational level, physical activity level, alcohol intake, height, weight, energy intake from fat source, energy intake from non-fat source, total saturated fat intake, total fibre intake, ever-use of postmenopausal hormones.
Harris, 2015 [33]	Swedish Mammography Cohort (SMC), Sweden	1987–2012, 15 years	1603; 37,004	Highest quartile vs lowest quartile; HR = 0.86 (0.72, 1.04)	Age, energy intake, height, BMI, education, oral contraceptive use, hormone replacement therapy use, age at menarche, age at menopause, family history of breast cancer, history of benign breast disease, smoking status, physical activity, alcohol intake.
Hashibe, 2015 [34]	Prostate, Lung, Colorectal and Ovarian (PLCO) Cancer Screening Trial, USA	1992–2011, 13 years (maximum)	1703; 50,563	≥2 cups/day vs. <1 cup/day; RR = 0.97 (0.87, 1.08)	Age, sex, race and education, drinking frequency.
Oh, 2015 [43]	Swedish Women’s Lifestyle and Health study, Sweden	1991–2012, 11 years (average)	1395; 42,099	≥5 cups/day vs. ≤2 cups/day; RR = 0.81 (0.70, 0.94)	Age, BMI, duration of breastfeeding, alcohol consumption.
Lukic, 2016 [40]	Norwegian Women and Cancer study (NOWAC), Norway	1991–2013, 13.1 years (average)	3277; 91,767	≥7 cups/day vs. ≤1 cup/day; HR = 0.87 (0.71, 1.06)	Menopausal status, smoking status, age at smoking initiation, number of pack-years, exposure to cigarette smoke during childhood, duration of education, BMI, physical activity level, alcohol consumption, number of children, age at first birth, ever use of oral contraceptives, duration of oral contraceptive use, use of hormone replacement therapy, maternal history of breast cancer, total energy intake, intake of fibres, intake of processed meat, intake of red meat, height, participation in mammography screening.

**Table 2:** Subgroup and additional analyses of studies reporting risk of breast cancer for the highest versus lowest (reference) category coffee consumption.

Subgroup	No. of Datasets	RR (95% CI)	*I*^2^	*P_heterogeneity_*
Total	16	0.96 (0.93, 1.00)	7%	0.37
(Alternative analysis)	18	0.96 (0.93, 1.00)	0%	0.48
Geographical location				
North America	7	0.97 (0.93, 1.02)	0%	0.84
Europe	7	0.95 (0.87, 1.05)	36%	0.15
Asia	2	1.01 (0.70, 1.47)	63%	0.10
Menopausal status				
Premenopausal	8	0.98 (0.89, 1.07)	0%	0.46
Postmenopausal	8	0.92 (0.88, 0.98)	0%	0.57
Receptor status				
ER+/PR+	5	0.97 (0.89, 1.07)	0%	0.40
ER+/PR- or ER-/PR+	5	0.98 (0.82, 1.17)	0%	0.60
ER-/PR-	5	0.92 (0.79, 1.07)	0%	0.82
Coffee type				
Caffeinated	6	0.96 (0.91, 1.01)	0%	0.45
Decaffeinated	6	0.97 (0.90, 1.04)	0%	0.67
BMI				
<25 kg/m^2^	5	0.98 (0.87, 1.10)	0%	0.42
≥25 kg/m^2^	5	0.91 (0.79, 1.04)	0%	0.69
Duration of follow-up				
<10 years	3	0.97 (0.80, 1.18)	0%	0.55
≥10 years	13	0.97 (0.92, 1.01)	20%	0.24
Adjustment for smoking				
No	7	0.95 (0.86, 1.05)	41%	0.12
Yes	9	0.97 (0.93, 1.01)	0%	0.67
Adjustment for alcohol intake				
No	5	1.02 (0.88, 1.19)	0%	0.41
Yes	11	0.96 (0.92, 1.00)	13%	0.32
Adjustment for physical activity				
No	10	0.97 (0.90, 1.04)	27%	0.20
Yes	6	0.96 (0.91, 1.01)	0%	0.58
Adjustment for education				
No	10	0.95 (0.87, 1.04)	32%	0.16
Yes	6	0.98 (0.93, 1.02)	0%	0.86

**Table 3:** Dose-response meta-analysis of prospective cohort studies on coffee consumption and breast cancer risk.

	No. of Datasets (No. of Studies)	Coffee Intake (Cups/Day)								*I*^2^ (%)	*P_heterogeneity_*	*P_non-linearity_*
		0	1	2	3	4	5	6	7			
Total												
Non-linear	13 (13)	Ref.	0.98 (0.94, 1.03)	0.97 (0.88, 1.06)	0.96 (0.84, 1.11)	0.96 (0.79, 1.16)	0.95 (0.75, 1.22)	0.95 (0.71, 1.28)	0.95 (0.67, 1.34)	-	0.69	0.52
Linear	13 (13)	Ref.	0.99 (0.98, 1.00)	0.98 (0.96, 0.99)	0.97 (0.94, 0.99)	0.96 (0.93, 0.99)	0.95 (0.91, 0.98)	0.93 (0.89, 0.98)	0.92 (0.88, 0.98)	-	0.58	NA
Postmenopausal												
Non-linear	6 (6)	Ref.	1.00 (0.96, 1.04)	0.99 (0.93, 1.05)	0.96 (0.89, 1.03)	0.92 (0.84, 1.00)	0.88 (0.79, 0.99)	0.85 (0.74, 0.97)	0.82 (0.69, 0.96)	0	0.71	0.14
Linear	6 (6)	Ref.	0.97 (0.95, 1.00)	0.95 (0.90, 1.00)	0.92 (0.86, 1.00)	0.90 (0.82, 0.99)	0.88 (0.78, 0.99)	0.85 (0.74, 0.99)	0.83 (0.70, 0.99)	39.6	0.14	NA
BMI > 25 kg/m^2^												
Non-linear	5 (5)	Ref.	0.98 (0.91, 1.06)	0.97 (0.86, 1.10)	0.97 (0.84, 1.12)	0.97 (0.82, 1.14)	0.97 (0.80, 1.18)	0.97 (0.76, 1.24)	0.97 (0.72, 1.30)	10.1	0.35	0,77
Linear	5 (5)	Ref.	0.99 (0.96, 1.02)	0.98 (0.92, 1.05)	0.97 (0.88, 1.08)	0.96 (0.84, 1.10)	0.95 (0.81, 1.13)	0.95 (0.77, 1.16)	0.94 (0.74, 1.19)	39	0.16	
ER-/PR-												
Non-linear	5 (4)	Ref.	0.93 (0.84, 1.04)	0.9 (0.76, 1.07)	0.91 (0.77, 1.08)	0.94 (0.79, 1.11)	0.97 (0.79, 1.19)	1.00 (0.77, 1.30)	1.04 (0.75, 1.43)	0	0.88	0.25
Linear	5 (4)	Ref.	0.99 (0.95, 1.03)	0.98 (0.91, 1.06)	0.97 (0.86, 1.10)	0.97 (0.82, 1.13)	0.96 (0.78, 1.17)	0.95 (0.75, 1.20)	0.94 (0.71, 1.24)	66.3	0.43	NA

## Supplementary Materials

The following are available online at http://www.mdpi.com/2072-6643/10/2/112/s1, Figure S1: Funnel plot for breast cancer risk of the highest versus lowest (reference) category of coffee consumption, Table S1: Meta-Analysis of Observational Studies in Epidemiology (MOOSE) checklist, Table S2: Search strategy.
